# Hospital-based clinicians lack knowledge and comfort in initiating medications for opioid use disorder: opportunities for training innovation

**DOI:** 10.1186/s13722-023-00386-x

**Published:** 2023-05-18

**Authors:** Andrea Jakubowski, Sumeet Singh-Tan, Kristine Torres-Lockhart, Shadi Nahvi, Melissa Stein, Aaron D. Fox, Tiffany Lu

**Affiliations:** 1 Department of Medicine, Division of General Internal Medicine, Albert Einstein College of Medicine/Montefiore Medical Center, 3300 Kossuth Avenue, Bronx, NY 10467 USA; 2 Department of Medicine, Hospital Division, Albert Einstein College of Medicine/Montefiore Medical Center, 111 E. 210 St, Bronx, NY 10467 USA

**Keywords:** Hospital-based MOUD initiation, Clinician education

## Abstract

**Background:**

Hospital-based clinicians infrequently initiate medications for opioid use disorder (MOUD) for hospitalized patients. Our objective was to understand hospital-based clinicians’ knowledge, comfort, attitudes, and motivations regarding MOUD initiation to target quality improvement initiatives.

**Methods:**

General medicine attending physicians and physician assistants at an academic medical center completed questionnaires eliciting barriers to MOUD initiation, including knowledge, comfort, attitudes and motivations regarding MOUD. We explored whether clinicians who had initiated MOUD in the prior 12 months differed in knowledge, comfort, attitudes, and motivations from those who had not.

**Results:**

One-hundred forty-three clinicians completed the survey with 55% reporting having initiated MOUD for a hospitalized patient during the prior 12 months. Common barriers to MOUD initiation were: (1) Not enough experience (86%); (2) Not enough training (82%); (3) Need for more addiction specialist support (76%). Overall, knowledge of and comfort with MOUD was low, but motivation to address OUD was high. Compared to MOUD non-initiators, a greater proportion of MOUD initiators answered knowledge questions correctly, agreed or strongly agreed that they wanted to treat OUD (86% vs. 68%, p = 0.009), and agreed or strongly agreed that treatment of OUD with medication was more effective than without medication (90% vs. 75%, p = 0.022).

**Conclusions:**

Hospital-based clinicians had favorable attitudes toward MOUD and are motivated to initiate MOUD, but they lacked knowledge of and comfort with MOUD initiation. To increase MOUD initiation for hospitalized patients, clinicians will need additional training and specialist support.

## Introduction

Medications for opioid use disorder (MOUD) are safe and effective. MOUD halves the risk of overdose mortality [1], reduces infectious complication of OUD, such as HIV and hepatitis C virus infection [2], and reduces emergency department use and hospital readmissions [3–5]. However, MOUD is greatly underutilized, with just 11% of people with OUD receiving MOUD annually [6]. Optimizing MOUD usage is a key strategy to improve the health of people with OUD and avoid preventable deaths, infections, and hospitalizations.

Targeting acute care settings for OUD diagnosis, treatment initiation, and referral to ongoing care is receiving greater attention in the United States [7]. OUD related hospitalizations, including for complications such as infectious endocarditis, have increased significantly in recent years [8]. Initiating MOUD in the acute care setting is feasible, effective and leads to improved engagement with outpatient treatment [9–11]. Among the three approved MOUDs in the US, opioid agonist medications—buprenorphine and methadone–have the strongest evidence base, and any licensed clinician can use them in hospitals without additional certification or training [12–14]. Ideally, hospital-based clinicians [i.e. hospitalists, general internists, physician assistants (PAs)] would diagnose OUD, offer MOUD during hospitalization, and facilitate linkage to ongoing treatment after discharge. However, despite the benefits of hospital MOUD initiation, hospital-based clinicians often miss opportunities to initiate these life-saving treatments [15].

Numerous clinician and system-level factors may contribute to MOUD underuse in hospitals. Clinicians lack training in substance use disorder treatment, including MOUD initiation [16]. Stigma toward OUD may reduce clinician willingness to master MOUD initiation [17]. Fractured healthcare systems make referring patients to outpatient MOUD treatment difficult. Few studies have directly examined why some hospital-based clinicians initiate MOUD, but having access to an addiction consult service may increase willingness to initiate MOUD [18]. Therefore, we sought to understand what factors most limited hospital-based clinicians in initiating MOUD.

Our objective was to identify clinician-level targets for quality improvement (QI) initiatives to increase hospital-based MOUD initiation. To do so, we evaluated clinicians’ knowledge, comfort, attitudes and motivations, because these are potentially modifiable barriers to MOUD initiation. We also explored whether clinicians who had initiated MOUD in the prior 12 months differed in knowledge, comfort, attitudes, and motivations regarding MOUD from those who had not. These findings may be useful to other health systems planning similar initiatives.

## Methods

We conducted a cross-sectional study in a large academic health center at the outset of a hospital-wide MOUD initiation QI project. In this study we: (1) determined hospital-based clinicians’ perceptions of barriers to MOUD initiation, and (2) evaluated hospital-based clinicians’ knowledge, comfort, attitudes, and motivations regarding OUD and MOUD initiation. These findings were used to inform targets for QI initiatives. The study was deemed exempt by the Albert Einstein College of Medicine Institutional Review Board.

### Study setting

Hospital-based clinicians were located at three academically-affiliated hospitals in Bronx, New York. The hospitals serve a patient population of mostly racially minoritized and publicly insured individuals and large number of patients with OUD; the largest of the three hospitals has over 160 admissions per month for OUD-related diagnoses. Two of the three hospitals have regular access to addiction consult services. Prior QI initiatives have developed standard protocols for hospital initiation of MOUD, but have not focused on training for hospital-based clinicians other than a small number of presentations at staff meetings.

### Study population

Hospital-based clinicians, including attending physicians and PAs, caring for patients on general medical services were invited to participate in the study. Inclusions were: (1) medical license eligibility to dispense buprenorphine and methadone in acute care settings (MD, doctor of osteopathic medicine (DO), nurse practitioner (NP), PA); (2) affiliation with one of the three hospitals; (3) completion of medical training (including residency for MDs and DOs); (4) spending any clinical time working clinically on the general medicine service.

### Participant recruitment

We recruited participants from June 14, 2021 to December 15, 2021 (6 months). We invited participants to complete the study during clinician meetings, and also publicized through flyers and email listservs across the three hospitals. To incentivize questionnaire completion, participants who completed the questionnaire received a $20 incentive.

### Data collection

We developed and administered an anonymous questionnaire to hospital-based clinicians. A hospitalist MOUD champion (SST), and addiction medicine specialists with expertise in OUD treatment (AJ, MS, KTL, SN, AF, TL) developed questionnaires. Domains were selected based on existing literature and expert opinions from collaborators on potential reasons why hospital-based clinicians may underutilize MOUD [18, 19]. Domains included hospital-based clinicians’ personal characteristics, perceived barriers to MOUD initiation, clinical knowledge of OUD and MOUD, comfort with MOUD initiation, clinical experience with OUD and MOUD, attitudes about OUD and MOUD, and motivation to address OUD. Questionnaires were administered anonymously using the Qualtrics platform and took approximately 5–10 min to complete.

### Measures

#### Clinician characteristics

We collected data on gender identity (male; female; transgender; other; decline to answer); clinician specialty (internal medicine; family medicine; other); clinician type (attending physician; PA; nurse practitioner); number of years since completing medical training (continuous); and percentage of clinical time spent in inpatient care (< 20%; 20–50%; 51–75%; > 75%), and hospital where clinicians practiced (choice of one of the three hospitals). We categorized participants as having access to an addiction consult service (two hospitals) or no access to an addiction consult service (one hospital).

#### Perceived barriers to initiating MOUD

Barriers to initiating MOUD were selected based on review of the literature and expert opinion [19–21]. We asked hospital-based clinicians about their level of agreement with statements about barriers to MOUD initiation (1 = strongly disagree, and 5 = strongly agree). Statements included one stem ("I may not initiate buprenorphine" or "I may not initiate methadone" because…) and a potential barrier (lack of time, training, experience, or nursing support). We dichotomized responses as agree or strongly agree vs. all other responses.

#### Prior 12-month MOUD initiation

We categorized participants as initiators or non-initiators based on self-reported initiation of buprenorphine or methadone during the prior 12 months for hospitalized patients that were not already receiving outpatient MOUD. We asked the number of patients for whom participants had initiated methadone and buprenorphine and created a dichotomous variable (0 = "non-initiators") and (≥ 1 = "initiators"). Participants only needed to report initiating either buprenorphine or methadone to be considered an MOUD initiator.

#### Other prior experience with MOUD

We asked participants whether they had ever completed a buprenorphine waiver training (waiver requirement was in effect during study period), prescribed buprenorphine to patients at discharge in the past 12-months, or had referred patients to buprenorphine or methadone treatment in the past 12 months.

#### Knowledge of OUD and MOUD

We developed questions with expert collaborators to identify knowledge gaps that might impact hospital MOUD initiation. We asked multiple-choice and true/false/unsure questions examining knowledge about methadone (three questions) and buprenorphine (three questions) (See Appendix 1). Answers were scored as correct or incorrect (unsure responses were considered incorrect).

#### Comfort with initiating MOUD

Participants were asked to indicate their level of comfort with skills necessary to initiate MOUD in hospital settings using a 5-point scale (1 = very uncomfortable, 5 = very comfortable). These skills included diagnosing opioid withdrawal and OUD, counseling patients on MOUD, initiating and titrating MOUD, writing a discharge prescription (buprenorphine only), and referring to outpatient MOUD treatment. We dichotomized responses as comfortable or very comfortable vs. all other responses.

#### Attitudes about OUD and MOUD

Participants indicated their agreement level with statements concerning attitudes about OUD (e.g. whether OUD is a choice, whether OUD is treatable) and MOUD (e.g. whether it is effective, whether patients are likely to continue MOUD after leaving the hospital) (1 = strongly disagree; 5 = strongly agree). These questions were designed to identify stigmatized attitudes and followed a previously published survey of general medicine clinicians [19]. We dichotomized responses as agree or strongly agree vs. all other responses.

#### Motivation to address OUD

We adapted questions from the Socrates motivation for change scale as well as other statements that may influence participants' motivation to address OUD (e.g. whether they frequently encounter OUD) [22]. There are no validated tools to measure clinicians' motivation to treat substance use disorders; therefore, we adapted the Socrates scale, which is typically used to assess motivation to change substance use behaviors, because it assesses both intentions to change and steps being taken to change behaviors. We dichotomized responses as agree or strongly agree vs. all other responses.

### Data analysis

Participants’ characteristics are described using medians, quartiles, frequencies, and percentages, where appropriate. First, we examined their perceived barriers to initiating MOUD and report the proportion agreeing or strongly agreeing with each statement. We also examined, using a Fisher's exact test, whether there was an association between identifying a need for more addiction specialist support (one of the barriers) and lacking access to an addiction consult service. Next, we evaluated knowledge of OUD and MOUD with a series of true/false and multiple choice questions and report proportion of correctly answered questions. For attitudes toward OUD and MOUD and motivation to address OUD, we report the proportion agreeing or strongly agreeing with each statement. Then, we examined self-reported MOUD initiation and report the proportion of clinicians who initiated MOUD in the prior 12 months. Finally, after dividing participants into initiators and non-initiators, we explored differences in prior buprenorphine waiver training completion, knowledge, attitudes, and motivation between the two groups using Chi-squared tests, Fisher’s exact, and Kruskal–Wallis tests, where appropriate. Given we had multiple items for each domain (6 items for Knowledge, 11 items for Comfort, and seven items each for Attitudes and Motivation), we calculated a Bonferroni-corrected alpha for each domain [(alpha of 0.05)/(number of items per domain)] to raise the threshold for statistical significance. Significant differences between initiators and non-initiators were considered to be key targets for QI initiatives.

Missing data: participant attrition occurred throughout the survey, with 174 participants beginning the survey and 143 completing it. Our primary analysis included the 143 participants who completed the survey. A sensitivity analysis that included all 150 participants who completed the questions about prior 12-month MOUD initiation (and thus could have been categorized as either initiators or non-initiators in main analyses), regardless of whether they completed the entire survey, did not demonstrate significant differences in main findings (data not shown).

Barriers section missing data: there was additional missing data in a single survey section: after starting data collection, we identified a skip pattern that had been erroneously programmed into Qualtrics, resulting in 40 participants skipping the barriers section of the survey. The error was such that participants who reported never having referred to methadone treatment at the academic medical center skipped the barriers section and advanced to the section on attitudes and beliefs. We evaluated whether there were differences in characteristics of participants who skipped the barriers section vs. those who did not using Chi-squared tests, Fisher’s exact, and Kruskal–Wallis tests where appropriate. We found that 35% of primarily outpatient clinicians skipped this section compared to 19% of primarily hospital-based clinicians (p = 0.024).

## Results

### Participant characteristics

Of the 143 participants who completed the survey, the majority were female (63%), internists (98%), and attending physicians (57%), spent greater than 50% time in the inpatient setting (69%), and completed their medical training within a median of 9 years [Q1–Q3] = [4–14]. The overall response rate based on the total number of clinicians who initiated the survey was 48%, while the data here represent 40%.

### Perceived barriers to initiating MOUD

**Fig. 1:**
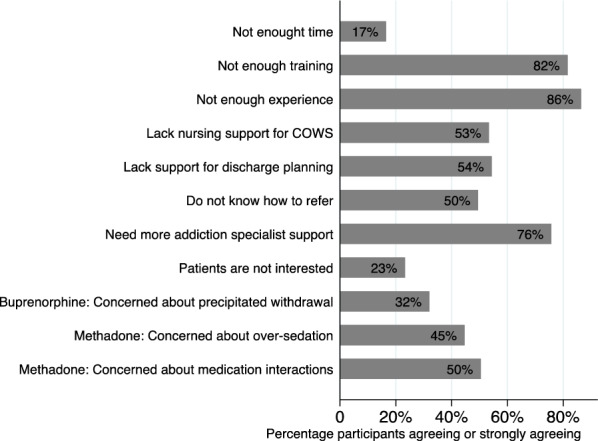
Perceived barriers to initiating MOUD (N = 103)

One-hundred and three participants completed the barriers section. The percentage of participants who agreed or strongly agreed with reasons they may not initiate methadone or buprenorphine are presented in Fig. 1.The most commonly reported reasons were not enough experience (86%), not enough training (82%), and need for more addiction specialist support (76%). Among participants with access to an addiction consult service, 74% reported needing more addiction specialist support, compared to 86% of those without access to addiction specialist support (p = 0.509). Fifty-four percent reported lacking support for discharge planning and 50% were unaware how to refer patients to outpatient MOUD treatment. Only 17% reported having insufficient time to initiate MOUD and 23% that patients were not interested in MOUD.

### Prior 12 month MOUD initiation

Fifty-five percent reported initiating MOUD for a hospitalized patient during the prior 12 months. Thirty-six percent had initiated buprenorphine, and 35% of participants initiated methadone. Characteristics of participants by prior 12-month MOUD initiation are presented in Table 1.

**Table 1:** Characteristics of participants among MOUD non-initiators and initiators

	Total	Non-Initiators n (%)	Initiators n (%)	p-value
Total	143 (100)	65 (100)	78 (100)	
Gender				0.343^a^
Male	51 (36)	22 (34)	29 (37)	
Female	90 (63)	41 (63)	49 (63)	
Decline to answer	2 (1)	2 (3)	0 (0)	
Clinician specialty				0.092^a^
Family medicine	3 (2)	3 (5)	0 (0)	
Internal medicine	140 (98)	62 (95)	78 (100)	
Clinician type				0.005^b^
Physician	82 (57)	29 (45)	53 (68)	
Physician assistant	61 (43)	36 (55)	25 (32)	
Years since completion medical training, median [Q1, Q3]	9 [4, 14]	10 [5, 14]	7 [4, 14]	0.207^c^
> 50% Time inpatient care	98 (69)	43 (66)	55 (71)	0.576^b^
Access to addiction consult service	120 (84)	55 (85)	65 (83)	0.835^b^
Completed buprenorphine waiver training	46 (32)	14 (21)	32 (41)	0.013^b^

^a^Fisher’s exact test

^b^Chi-squared test

^c^Kruskall-Wallis test

### Other prior experience with MOUD

Forty-three percent had referred to outpatient buprenorphine treatment, and 48% had referred to outpatient methadone treatment. Thirty-two percent of participants had completed buprenorphine waiver training, and of these, 78% obtained their DEA-X number to prescribe buprenorphine. Compared to MOUD non-initiators, a greater percentage of MOUD initiators had completed a buprenorphine waiver training (41% vs. 22%, p = 0.013).

### Differences between initiators and non-initiators

Among those who had initiated buprenorphine and/or methadone, the median number of patients initiated was 2 [Q1, Q3] = [1, 2] for buprenorphine and 3 [Q1, Q3] = [1, 5] for methadone. Compared to MOUD non-initiators, a greater proportion of MOUD initiators were physicians (68% vs. 32%, p = 0.005) and had fewer years since completing medical training (7 [Q1, Q3] = [4, 14] vs. 10 [Q1, Q3] = [5, 14], p = 0.207).

### Knowledge of OUD and MOUD

Overall, knowledge was limited, particularly about the legality of initiating and titrating MOUD in the hospital setting and MOUD safety. Differences in knowledge between MOUD initiators and MOUD non-initiators are presented in Table 2. The Bonferroni-corrected alpha for the knowledge section was 0.008. Compared to MOUD non-initiators, more MOUD initiators answered correctly that buprenorphine can be used to treat withdrawal (89% vs. 77%, p = 0.066), that a buprenorphine waiver was unnecessary to administer buprenorphine to hospitalized patients, (51% vs. 32%, p = 0.022) and that concurrent use of antiepileptic drugs, QT-prolonging agents and benzodiazepines were not absolute contraindications to administering methadone (60% vs. 34%, p = 0.002).

**Table 2:** Knowledge of and Comfort with OUD and MOUD among MOUD non-initiators and initiators

	Total (N = 143)	Non-Initiators n (%) N = 65	Initiators n (%) N = 78	p-value^a^
Knowledge: proportion participants answering correctly				
Bupe^b^ can be used to treat withdrawal	119 (83)	50 (77)	69 (89)	0.066
Bupe ceiling effect	92 (64)	34 (52)	58 (74)	0.006^c^
Bupe waiver required to dispense in bupe in hospital	61 (43)	21 (32)	40 (51)	0.022
Methadone medication interactions	69 (48)	22 (34)	47 (60)	0.002^c^
Methadone legality of dispensing > 30 mg	56 (39)	25 (38)	31 (40)	0.876
Changing methadone dose for patients enrolled in program	72 (50)	29 (45)	43 (55)	0.211
Comfort: proportion participants "comfortable" or "very comfortable" with				
Diagnosing opioid withdrawal	81 (57)	36 (55)	45 (58)	0.782
Diagnosing OUD	82 (57)	33 (51)	49 (63)	0.147
Counseling about bupe	47 (33)	17 (26)	30 (38)	0.119
Initiating bupe	25 (17)	9 (14)	16 (21)	0.296
Titrating bupe	35 (24)	14 (22)	21 (27)	0.456
Writing a bupe discharge prescription	32 (22)	10 (15)	22 (28)	0.067
Referring to bupe	87 (61)	41 (63)	46 (59)	0.617
Counseling about methadone	70 (49)	26 (40)	44 (56)	0.051
Initiating methadone	39 (27)	10 (15)	29 (37)	0.004^d^
Titrating methadone	36 (25)	14 (22)	22 (28)	0.360
Referring to methadone	102 (71)	46 (71)	56 (72)	0.893

^a^All p-values are for Chi-squared tests

^b^Bupe: Buprenorphine

^c^Significant at Bonferroni-corrected alpha of 0.008

^d^Significant at Bonferroni-corrected alpha of 0.005

### Comfort with OUD and MOUD

In the entire sample (N = 143), 57% were comfortable or very comfortable diagnosing opioid withdrawal and 57% were comfortable or very comfortable diagnosing OUD. Overall, comfort with MOUD was low, but higher for methadone than buprenorphine. Only 33% were comfortable or very comfortable counseling patients about buprenorphine compared to 49% for methadone. Only 17% were comfortable or very comfortable initiating buprenorphine compared to 27% for initiating methadone. The only significant difference between MOUD initiators and non-initiators was that a greater proportion of MOUD initiators reported being comfortable with initiating methadone compared to non-initiators (37% vs. 15%, p = 0.004; Bonferroni-corrected alpha = 0.005).

### Attitudes about OUD and MOUD

Overall, attitudes were favorable toward OUD and MOUD, with few clinicians agreeing with stigmatizing statements. Few differences in attitudes between MOUD initiators and MOUD non-initiators were noted (Table 3). The Bonferroni-corrected alpha for the attitudes section was 0.007. In particular, 90% of MOUD initiators agreed or strongly agreed that treatment of OUD with medication is more effective than without medication, compared to 75% of MOUD non-initiators (p = 0.022). Forty-six percent reported that caring for patients with OUD is as professionally satisfying as other activities.

**Table 3:** Attitudes and motivation toward OUD and MOUD among MOUD non-initiators and initiators

	Total	MOUD non-initiators n (%) N = 65	MOUD initiators n (%) N = 78	p-value^a^
Attitudes: proportion participants "agreeing" or "strongly agreeing" with				
OUD is a choice	13 (9)	8 (12)	5 (6)	0.222
MOUD is replacing one addiction with another	17 (12)	9 (14)	8(10)	0.509
Caring for patients with OUD is as satisfying as other activities	66 (46)	26 (40)	40 (51)	0.178
OUD is a treatable disease	122 (85)	55 (85)	67 (86)	0.829
Treatment of OUD with medication is more effective than without	119 (83)	49 (75)	70 (90)	0.022
It is not the role of the hospital clinician to start MOUD	13 (9)	7 (11)	6 (8)	0.524
Patients are unlikely to continue treatment	17 (12)	9 (14)	8 (10)	0.509
Motivation: proportion participants "agreeing" or "strongly agreeing" with				
I really want to address OUD among my patients	111 (78)	44 (68)	67 (86)	0.009
I have already started addressing OUD among my patients	86 (60)	28 (43)	58 (74)	0.000^b^
I have worked to increase my knowledge of OUD	93 (65)	36 (55)	57 (73)	0.027
Not addressing OUD negatively impacts my patients	110 (77)	49 (75)	61 (78)	0.690
OUD is a problem I encounter often	105 (73)	39 (60)	66 (85)	0.001^b^
I already use MOUD	65 (45)	20 (31)	45 (58)	0.001^b^
I already use MOUD and want to learn more	87 (61)	33 (51)	54 (69)	0.024

^a^All p-values are for Chi-squared tests

^b^Significant at Bonferroni-corrected alpha of 0.007

### Motivation to address OUD

Overall, motivation was high. Differences in motivation to address OUD between MOUD initiators and MOUD non-initiators are presented in Table 3. The Bonferroni-corrected alpha for the motivation section was 0.007. Greater differences were found between groups in motivation compared to other domains. Compared to MOUD non-initiators, a greater proportion of MOUD initiators agreed or strongly agreed that they really wanted to address OUD among their patients (86% vs. 68%, p = 0.009), that OUD is a problem they encounter often (85% vs. 60%, p = 0.001), and that they had worked to increase their knowledge of OUD (73% vs. 55%, p = 0.027).

## Discussion

We identified key targets for future QI initiatives by examining hospital-based clinician’s knowledge, comfort, attitudes, and motivations regarding MOUD initiation. Overall, clinicians lacked knowledge of and comfort with OUD and MOUD, despite reporting positive attitudes and high motivation to address OUD. While most had initiated some form of MOUD in the past 12 months, experience was minimal and limited to only one type of MOUD. Prior 12-month MOUD initiation was associated with clinician knowledge, comfort, attitudes and motivation. Importantly, while most hospital-based clinicians were comfortable with basic tasks, such as diagnosing opioid withdrawal and OUD and referring to outpatient MOUD, few were comfortable with more advanced tasks, such as *initiating* and managing MOUD. These gaps between knowledge and comfort among otherwise motivated clinicians are key targets for QI interventions in MOUD initiation.

Low rates of MOUD initiation were striking given the hospitals’ rollout of detailed work-flows, clinicians' positive attitudes toward OUD and MOUD and high motivation to address OUD. We expected that stigma would manifest as negative attitudes toward OUD, as reported in prior studies. In a 2022 study of hospitalists regarding OUD-related care, Calcaterra et al. found that a greater proportion of providers agreed or strongly agreed that addressing OUD is not the role of the hospital provider than in our study (29% vs. 9%) [18]. In a different 2016 study of hospitalists, 38% reported that OUD was a choice and only 38% reported finding substance use disorder care as satisfying as other clinical activities [19]. Our study found the opposite, with few clinicians agreeing with stigmatizing statements such as "addiction is a choice" and " MOUD is replacing one addiction with another" [18]; even though fewer than half agreed that caring for patients with OUD was as satisfying as other clinical activities. Since 2016, opioid overdoses have increased dramatically, which has likely led to greater awareness and knowledge about OUD and its treatments among hospital-based clinicians [23, 24]. The incongruity between positive attitudes/motivations and modest MOUD initiation and low satisfaction with providing OUD care may indicate uncaptured stigma among clinicians (i.e., social desirability bias in reporting attitudes), but it also may reflect frustration from knowing that they *should* provide high-quality OUD care, while they lack the training and experience to do so. A study by Englander et al. found that hospital-based clinicians experienced “moral distress” if they lacked the knowledge, comfort and support to effectively care for patients with substance use disorders [25]. Future qualitative research could explore the discrepancy between positive attitudes and modest MOUD initiation and low satisfaction further.

Unlike barriers to MOUD initiation previously described by clinicians in emergency departments, we found that practitioners in hospital settings did not identify the lack of time and lack of patient interest as key barriers [20, 21]. Instead, clinicians reported lack of training and experience as the most important barriers, followed by lack of support at the systems level (i.e. lack of addiction specialty, nursing, discharge planning support). These modifiable barriers underline the need for both clinician-level interventions to increase training and experience and systems-level interventions to provide the infrastructure and support required for MOUD initiation. Systematic screening for and evaluation of opioid withdrawal could prompt clinicians to initiate MOUD and support them in appropriate MOUD titration [26]. In the ED setting, recruitment of clinician champions, frequent reminders, standardized scripts for communicating with patients, and streamlined protocols have supported clinicians to initiate MOUD [27–29]. Peer recovery coaching or patient navigation programs may assist hospital-based clinicians in linking patients to outpatient MOUD treatment, addressing the lack of support for and knowledge of discharge planning they reported in our study [30, 31]. Implementing specialized addiction consult services would also support MOUD initiation and linkage to outpatient MOUD treatment [32]. Our finding that clinicians who had access to addiction consultative services still identified a need for more addiction specialist support was surprising, and likely reflects variability in consult service availability (i.e. one hospital in our study only had consultant availability a few days per week). Ultimately, many hospitals will lack the resources and addiction expertise needed to establish full-time consultative services, underlining the importance of training hospital-based clinicians in MOUD initiation.

Our finding that clinicians’ attitudes and motivation were associated with MOUD initiation emphasizes that individual clinicians play a critical role in determining whether patients receive evidence-based OUD treatment. Encouragingly, MOUD initiators *and* non-initiators had positive attitudes and high motivation toward MOUD initiation, indicating possible receptiveness to training interventions that enhance competency in OUD care. The most effective content, structure, and modality of these interventions is an open question*.* However, based on our data on lack of knowledge and comfort, training hospital-based clinicians in state and federal regulations allowing for hospital-based MOUD initiation and titration, emphasizing the safety and effectiveness of MOUD, and providing case-based practice with counseling patients about MOUD as well as MOUD initiation and titration protocols could potentially increase MOUD initiation. We have developed an interactive, small group training in MOUD initiation for hospital-based clinicians supported by one-on-one coaching from a clinical champion, which may promote building motivation, confidence, and skill. Expanding the skills of hospital-based clinicians in OUD management could address key gaps in care, particularly in settings without or with limited addiction consult services.

We also found that physicians in our sample were more likely than physician assistants (PAs) to have initiated MOUD. PAs may receive less training in OUD management than MDs. Still, PAs (and NPs) have reported high interest in prescribing buprenorphine and represent important increases in buprenorphine waivered providers in the US, particularly in rural areas [33, 34]. Thus, efforts to provide training in MOUD initiation to hospital-based practitioners should be tailored towards PAs and NPs, as well as physicians.

Our study’s strengths include that we sampled hospital-based clinicians from three hospitals, some with and others without addiction consultants, increasing the generalizability of our findings. We also included PAs, where previous studies have focused exclusively on hospitalist physicians. Finally, we asked clinicians about methadone initiation, where prior studies have focused exclusively on buprenorphine. The study’s limitations include: the cross-sectional study design, which limits causal inferences regarding knowledge, comfort, attitudes, motivation and MOUD initiation; missing data in the perceived barriers section, potentially limiting the generalizability of this section; and reliance on self-report of prior 12 month MOUD initiation, which could contribute to inaccuracies.

## Conclusions

In conclusion, we found an important gap between clinician attitudes and motivation and self-reported MOUD initiation behavior. Hospital-based clinicians are motivated to initiate MOUD and should be supported to do so through trainings and opportunities to gain confidence and experience, while systems-level changes also reduce barriers to MOUD initiation. Improving hospital clinician knowledge, comfort and skill in MOUD initiation is a crucial step to improving access to evidence-based OUD treatment.

## Data Availability

The datasets used and/or analyzed during the current study are available from the corresponding author on reasonable request.

## Abbreviations

OUD: Opioid use disorder
MOUD: Medications for OUD
Bupe: Buprenorphine
PA: Physician assistant
NP: Nurse practitioner
QI: Quality improvement
